# Impact of Acute (Poly)Phenol-Rich Sugarcane Extract Consumption on Postprandial Glycemic Response in Healthy Adults: A Randomized Crossover Study

**DOI:** 10.3390/foods15040631

**Published:** 2026-02-10

**Authors:** Ulluwis H. A. J. Hewawansa, Elizabeth Barber, Michael J. Houghton, Rizliya Visvanathan, Luca Nicolotti, Ricardo J. S. Costa, Gary Williamson

**Affiliations:** 1 Department of Nutrition, Dietetics and Food, School of Clinical Sciences at Monash Health, Faculty of Medicine, Nursing and Health Sciences, Monash University, 264 Ferntree Gully Road, Notting Hill, VIC 3168, Australia; ulluwis.hewawansa@monash.edu (U.H.A.J.H.); elizabeth.barber@monash.edu (E.B.); michael.houghton@monash.edu (M.J.H.); rizliya.visvanathan1@monash.edu (R.V.); ricardo.costa@monash.edu (R.J.S.C.); 2 Victorian Heart Institute, Monash University, Victorian Heart Hospital, 631 Blackburn Road, Clayton, VIC 3168, Australia; 3 The Australian Wine Research Institute, P.O. Box 46, Glenside, SA 5065, Australia; luca.nicolotti@awri.com.au; 4 Metabolomics Australia, P.O. Box 46, Glenside, SA 5065, Australia; 5 School of Biological Sciences, Queen’s University, 19 Chlorine Gardens, Belfast BT9 5DL, UK

**Keywords:** human, glucose, insulin, Matsuda index, HOMA-IR, α-glucosidase, α-amylase, phenolic acid, flavonoids, polyphenol

## Abstract

**Background:** Effects on insulin sensitivity and postprandial glycemia through enzyme inhibition and regulation of glucose transport have been extensively researched; however, the role of sugarcane (poly)phenols remain underexplored. **Methods:** In a randomized, placebo-controlled, single-blinded crossover study, 12 healthy participants consumed a bread-based meal containing 50 g of carbohydrates, supplemented with either 0.5% or 5% liquid PRSE or sugar-balanced controls. Glucose and plasma insulin levels were assessed over 180 min. The extract was evaluated for its inhibitory effect on human α-amylases (salivary and pancreatic) and α-glucosidases (sucrase, maltase, and isomaltase) utilizing solid PRSE. **Results:** The postprandial glucose and insulin responses to bread sandwiches in healthy volunteers remained unchanged by both PRSE dosages. High-dose treatment reduced the Matsuda index by 9.8%, perhaps due to a subtle alteration in whole-body insulin sensitivity. Low-dose intervention postponed the insulin peak by 30 min without altering HOMA-IR. In vitro, PRSE diminished sucrase activity by 67% (IC_50_ = 425.8 ± 18.7 µg/mL) and lowered maltase and isomaltase activity by 40% (IC_25_ = 876.3 ± 131 and 960.6 ± 95.2 µg/mL, respectively). It enhanced the activity of human salivary and pancreatic α-amylases. **Conclusion:** In healthy people, acute PRSE supplementation had a minor impact on postprandial glucose and insulin levels. Low-dose PRSE postponed the insulin peak, whereas high-dose PRSE reduced Matsuda index potentially via α-amylase activation, suggesting a modest alteration in whole-body insulin sensitivity without significantly changing the glucose or insulin response. In vitro, PRSE exhibited modest inhibition of human α-glucosidases.

## 1. Introduction

There is a major shift in current research towards the use of dietary interventions as preventive strategies to improve insulin sensitivity and glycemic control, driven by the global rise in metabolic diseases, including type 2 diabetes [1]. Among these dietary elements, (poly)phenols, natural bioactives from plant-based foods and beverages, have been extensively explored for their potential in modulating glucose metabolism [2]. Sugarcane is rich in (poly)phenols, primarily flavonoids (apigenin, luteolin, and tricin) and phenolic acids (ferulic acid, caffeic acid, and their precursors, chlorogenic acids) as reviewed earlier [3].

Apigenin has been shown to protect pancreatic β-cells and facilitate GLUT4 translocation in skeletal muscles of streptozotocin-induced diabetic rats, thereby enhancing glucose homeostasis [4]. Similarly, caffeic acid (at 10–100 mg/kg/day) decreased blood glucose levels of streptozotocin- or alloxan-induced diabetic rats [5]. Likewise, chronic intake of 400 mg green coffee bean extract (rich in chlorogenic acid) twice daily for 8 weeks reduced fasting blood glucose (*p* = 0.03) and the homeostatic model assessment of insulin resistance (HOMA-IR) score (*p* = 0.02) in participants with metabolic syndrome [6].

While sugar from sugarcane (*Saccharum officinarum*) is widely consumed as a ubiquitous sweetening agent in food, (poly)phenolic compounds derived from sugarcane extracts have been underexplored for their biological effects. (Poly)phenol-rich sugarcane extract (PRSE) is a resin-based extract derived from sugarcane molasses (a by-product of sugarcane processing). Limited evidence suggests that PRSE may reduce glucose absorption in both sodium-dependent and independent pathways and restore insulin synthesis in functionally impaired β-cells in vitro [7]. However, their effects on carbohydrate-digesting enzymes (crucial steps in digestion) in humans are entirely unknown. The limited preliminary clinical data reported a dose-dependent 27–42% reduction in area under the curve (AUC) of blood glucose response curves after an acute intake of 19–32 mg PRSE/100 g of sugar (*p* < 0.05) compared with control (50 g glucose), following a sucrose challenge during breakfast [8]. (Poly)phenols potently influence postprandial glycemic responses through several mechanisms, including inhibition of carbohydrate-digesting enzymes (α-amylases and α-glucosidases), suppression of intestinal glucose transporters, stimulation of insulin secretion from pancreatic β-cells, and reduction in hepatic glucose production [9,10].

To address the limitations in clinical data, we conducted a randomized, placebo-controlled, crossover study to evaluate the effects of two doses of PRSE on postprandial glucose and insulin responses following a standardized sandwich meal challenge in healthy individuals. Further, the inhibitory potential of PRSE on human α-amylase and α-glucosidase activities was tested using our previously validated enzyme assays [11].

## 2. Materials and Methods

Substrates and standards (glucose, fructose, sucrose, maltose, and isomaltose), acetonitrile, human salivary and pancreatic α-amylases, Folin–Ciocalteu reagent, methanol, chloroform, gallic acid, and other solvents/reagents were acquired from Merck Life Science (Bayswater, VIC, Australia). Purity was above 98%. Cell culture media, phosphate buffer solution (PBS), trypsin, and the Pierce Coomassie (Bradford) assay kit were supplied by Thermo Fisher Scientific (Scoresby, VIC, Australia). For all preparations and solutions, Milli-Q water (18.2 MΩ/cm) was used. High-performance anion-exchange chromatography with pulsed amperometric detection (HPAEC-PAD) was used to detect and quantify sugars using the Dionex^TM^ Integrion^TM^ HPIC^TM^ (Thermo Fisher Scientific, Scoresby, VIC, Australia) system. Maltoheptaose (Mal-7; purity > 90%, Megazyme International, Bray, Ireland) was used as the substrate for the human α-amylase assays. A PHERAstar FS^TM^ plate reader (BMG Labtech, Ortenberg, Germany) was used to quantify absorbance in the Bradford total protein assay.

For the human study, a (poly)phenol-rich sugarcane extract was supplied by The Product Makers (Keysborough, VIC, Australia). White sandwich bread was purchased from Coles Supermarkets Australia Pty Ltd. (Hawthorn East, VIC, Australia). Woolworths ricotta light cheese and Lurpak unsalted butter were purchased from Woolworth Supermarkets Australia (Woolworths Group, Bella Vista, NSW, Australia). Blood sample collection microcuvettes (Hemocue Glucose 201 RT) and HemoCue^®^ Glucose 201 RT System were purchased from Radiometer Pacific Pty Ltd. (Mount Waverley, VIC, Australia). The Unistik 3 Extra 21G 2.0 mm lancets were purchased from Medshop, Dandenong South, VIC, Australia. Blood samples for insulin analysis were collected into Safe-T-Fill™ Capillary Blood Collection tubes (GK Systems containing EDTA anti-coagulant) purchased from Kabe Labortechnik GmbH (Nümbrecht, Germany) and separated using the Eppendorf Centrifuge 5702 R (Eppendorf AG, Hamburg, Germany), where plasma was collected into the 1.7 mL microcentrifuge tubes (Eppendorf AG, Hamburg, Germany). The Millipore ELISA kit for human insulin (Cat. #EZHI-14K, Millipore, Burlington, MA, USA) was purchased from Abacus Dx, Cannon Hill, Queensland, Australia. The Quintron BreathTracker Digital Microlyzer (QuinTron Instrument Company Inc., New Berlin, WI, USA) was used to analyze the breath samples collected in breath bags.

For (poly)phenol extraction, Strata-X solid phase extraction cartridges (Phenomenex Australia, Lane Cove, New South Wales, Australia), followed by centrifugal evaporation was used. For (poly)phenol profile analysis a Thermo Fisher Orbitrap IDX FTMS mass spectrometer (Thermo Fisher Scientific, Scoresby, VIC, Australia) was used.

### 2.1. Human Study

**Figure 1:**
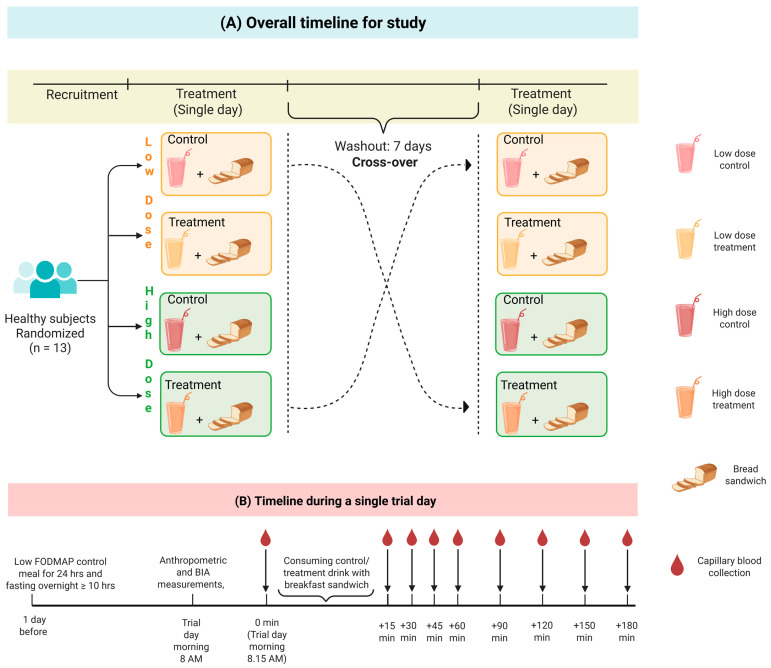
Schematic flow diagram of overall study design (**A**), detailed trial day experimental protocol and sample collection times (**B**). FODMAP: Fermentable oligosaccharide, disaccharide, monosaccharide, and polyols; BIA: Body impedance analysis. Blood glucose was measured at each timepoint shown, while blood samples collected for plasma insulin measurement were collected at every 30 min interval pre- and post-consumption on the trial day. Created in BioRender. Hewawansa, U. (2026) https://BioRender.com/5fm69ds.

A single-blinded, placebo-controlled, randomized crossover study was performed on healthy individuals to assess the effects of PRSE (Figure 1). The study was registered with the Australian New Zealand Clinical Trials Registry (ACTRN12624000242527, https://www.anzctr.org.au/ACTRN12624000242527.aspx, accessed on 3 February 2026), and ethics approval was granted by the Monash University Human Research Ethics Committee (MUHERC#34576). All procedures were carried out in accordance with the Helsinki Declaration, and written consent was obtained from all participants. No interim analyses were planned.

#### 2.1.1. Participant Recruitment and Screening

Participants were recruited via advertising flyers and social media. The initial screening process involved completing a comprehensive questionnaire in Qualtrics Survey software (Qualtrics XM, Provo, UT, USA). This questionnaire was used to collect demographic information and to exclude individuals who did not meet the inclusion criteria. Inclusion criteria for participation in the study were age 18 years or older, fasting blood glucose levels within the range of 3.9–5.5 mmol/L, and without intake of any medications that may affect blood sugar (i.e., metformin). Exclusion criteria encompassed gastrointestinal conditions, health conditions that could interfere with participation, such as smoking history, recent major surgery, implanted cardiac defibrillator, pregnancy, planning to become/currently pregnant, or breastfeeding.

During the screening visit, participants visited the Monash University BASE Facility (Notting Hill, VIC, Australia) after ~10 h fasting overnight and refrained from engaging in strenuous exercise for 24 h where their height, weight, waist circumference, body composition, blood pressure, and a fasting finger-pricked blood sample was collected to measure glucose concentration for initial assessment and insulin concentration later. Participants with fasting blood glucose levels between 3.9 and 5.5 mmol/L were selected for the study, while those with levels exceeding 7.0 mmol/L were advised to consult their general practitioner. Participants also completed questionnaires to assess physical activity levels.

#### 2.1.2. Randomization and Blinding

Randomization of the sequence in which the interventions and controls were provided to participants was performed by another investigator (EB) using a Latin square design. Participants were provided with the intervention or control drinks in opaque bottles, and the meal was served in a private space during each visit in a random order, blinded to the main investigator (UHAJH), who conducted blood sampling, data collection, and analysis. This study was single-blinded, as participants could identify the taste difference between the drinks, and they were not informed which was the test drink.

#### 2.1.3. Interventions and Controls

The clinical intervention used PRSE in liquid form (PRSE-L), which is an ethanolic resin-based extraction of molasses, a by-product of the sugarcane industry. It has 44.2% sugars (32% sucrose, 6.9% fructose, 5.3% glucose). The low-dose (LD of 0.5% *w*/*w*) and high-dose (HD of 5% *w*/*w*) were prepared by mixing PRSE with water (LD: 1 g PRSE-L + 199 g water; HD: 10 g PRSE-L + 190 g water). The control samples were prepared to match the sugar concentrations of each dose (LD: 0.16% sucrose, 0.04% fructose, 0.03% glucose HD: 1.6% sucrose, 0.35% fructose, 0.27% glucose). The bread sandwich contained 50 g of carbohydrates, formulated using Foodworks Professional v2 (Foodworks.online, Brisbane, QLD, Australia), consisting of white bread (103.4 g), butter (5 g) and ricotta cheese (15 g). To reduce variability between treatments, the sandwich was prepared in bulk, frozen at −20 °C for convenience, and thawed at 4 °C the day before each visit.

#### 2.1.4. Treatment Sessions

Four treatment sessions were scheduled for eligible participants at the BASE Facility with at least a 7-day washout period after the ~10 h fasting period before each session. Participants were provided with a 24 h low-FODMAP (Fermentable oligosaccharides, disaccharides, monosaccharides and polyols) diet before their visits to eliminate any confounding dietary variables induced by microbial differences. The diet included low-FODMAP certified cereal and lactose-free milk for breakfast, meals from We Feed You Pty Ltd. (Footscray, VIC, Australia) for lunch and dinner and low-FODMAP snacks at other times. Participants were instructed to avoid strenuous exercise for 24 h before each visit.

During each treatment session, participants’ height, weight, waist circumference, body composition, and blood pressure readings were collected. Fasting finger-prick blood samples for glucose and insulin were collected immediately before the meal intervention. In addition, breath samples were collected to confirm compliance with the low-FODMAP diet and to monitor carbohydrate malabsorption during the trial. Breath samples were analyzed for hydrogen (with 20 ppm cut-off) and methane (with 10 ppm cut-off) levels [12]. The bread sandwich was served along with one of the treatment drinks and a 250 mL bottle of water. Timers were started upon the first bite of the meal, and the participants were asked to finish the whole meal within 10 min after starting the timer. Participants were advised to alternate between the test drink and the bread sandwich. Participants were asked to consume the 250 mL water bottle throughout the study. Finger-prick blood samples and breath samples were collected at 15 min intervals during the first hour after the meal and every 30 min for the next 2 h. Postprandial blood samples for insulin were only taken every 30 min (Figure 1) using 200 µL Safe-T-Fill^TM^ capillary blood collection tubes. The capillary tubes were centrifuged for 10 min at 3 RCF at 4 °C and the plasma samples were stored at −80 °C until further analysis.

Plasma insulin concentrations were measured in duplicate from 20 µL samples using Millipore ELISA Kits for Human Insulin according to the instructions in the kit, and absorbances were measured at 370 nm, with detection limits ranging from 1.0 to 200 µU/mL. Samples exceeding the detection limit were diluted 2:1 in the assay buffer and reanalyzed.

Using insulin and glucose levels, the HOMA-IR score [13] and Matsuda index (MI) [14] were calculated using the following formulas.

(1) $$HOMA-IR={Insulin}_{basal}\;\left[\frac{\mu IU}{mL}\right]\times {Glucose}_{basal}\;\left[\frac{mmol}{L}\right]÷22.5$$

(2) $$Matsuda\;index=10,000÷\surd ({Glucose}_{basal}\;\left[\frac{mg}{dL}\right]\times {Insulin}_{basal}\;\left[\frac{\mu IU}{mL}\right]\times \;\;{Glucose}_{mean}\;\left[\frac{mg}{dL}\right]\times {Insulin}_{mean}\;\left[\frac{\mu IU}{mL}\right])$$

### 2.2. Human α-Glucosidase Enzyme Inhibition Assay

The immortalized human colon adenocarcinoma-derived Caco-2/TC7 cells (RRID: CVCL_0233) were a kind donation from the Rousset Lab (INSERM U505, Paris, France) [15]. After achieving 100% confluence, the cells underwent 21 days of differentiation with medium changes every 2–3 days. The cultured cell-free extracts (CFEs) were prepared for α-glucosidase enzyme assays using our validated method [11], and the total protein content of the CFEs was determined by the Bradford assay [16].

The α-glucosidases from Caco-2/TC7 cells exhibit high sucrase, maltase, and isomaltase activities, making them the most suitable in vitro candidates for assessing the disaccharide digestive mechanism in humans. The substrates sucrose, maltose and isomaltose were hydrolysed in the presence of the inhibitors, including PRSE (100–2000 µg/mL), and were compared with and corrected for negative controls and blanks (substrate, CFE and/or PRSE alone). Substrates, CFEs, and PRSE were prepared similarly in phosphate saline buffer (PBS, pH 6.9). The enzyme assay protocol and the method for determining sugar concentration by HPAEC-PAD using standard curves are detailed in our published protocol [11].

### 2.3. Human Salivary and Pancreatic α-Amylase Inhibition Assays

Human salivary and pancreatic α-amylase inhibition assays were performed similarly using commercially available enzymes (Merck Life Science, Bayswater, VIC, Australia) and Mal-7 as the substrate, following the previously reported method with quantification of sugar products by HPAEC-PAD [11,17].

### 2.4. Analysis of Total (Poly)Phenol Content and (Poly)Phenolic Profile in PRSE

PRSE was used in two different forms during this research study. Human intervention was conducted using the PRSE-L form, a resin-based extraction of molasses. All enzyme studies were conducted using PRSE-S, a form of PRSE-L that has been further processed to remove excess sugar and moisture, as the presence of sugar can introduce misinterpretation of enzyme inhibition results. Total (poly)phenol content of PRSE-L was measured using the Folin–Ciocalteu assay using gallic acid as a standard, and the values are reported as gallic acid equivalents (GAE). (Poly)phenol extraction was carried out as previously described [18]. Liquid chromatography-mass spectrometry (LC-orbitrap-MS, Scoresby, VIC, Australia) was used to identify the (poly)phenols in both PRSE-L/S, as previously described [18]. The relative peak area was reported as normalized to weight and compared to the relatively stable compound (poly)phenol 3-*O*-feruloylquinic acid (3FQA).

### 2.5. Statistical Analyses

This study’s sample size was based on prior investigations, which found a statistically significant glycemic and insulinemic difference in 10 subjects [19,20,21]. The article on the glycemic index technique [22] suggests a minimum of 10 participants for investigations on human glycaemic response provides reasonable degree of power and precision. Assuming a 20% drop out/missing data rate, the sample size was determined as 12. Thirteen participants were recruited. Participants with missing data points (n = 1) were excluded and the analyses were only performed using complete case data. Since a 20% drop out/missing data rate was previously accounted for, this exclusion did not impact statistical power. Statistical analyses were performed using IBM SPSS Statistics 30.0.0.0 (Chicago, IL, USA). Data visualization of figures and graphs were performed using GraphPad 10.2.0 (Boston, MA, USA). A schematic flow diagram of the study was created by Biorender (biorender.com). Data are presented as mean ± standard error of the mean (SEM). Incremental area under the curve (iAUC) was calculated by correcting each individual’s blood glucose levels for baseline levels. Peak blood glucose/insulin was determined using mean plot peaks. Peak time was defined for individual subject in a pre-specified time frame (0–180 min). If two peaks occurred, earliest occurrence was considered as the peak. The Shapiro–Wilk test and a visual examination of residual plots were used to determine whether the data were normally distributed. The impact of the two interventions relative to controls was measured using paired sample *t*-tests, two-factor repeated-measures analysis of variance (ANOVA) with Tukey’s post hoc comparisons and a 95% confidence interval. iAUC, peak blood glucose/insulin concentrations, MI, and the HOMA-IR score between the four groups were deemed significant at *p* < 0.05.

## 3. Results

### 3.1. Participant Characteristics and Nutritional Composition of the Breakfast Sandwich

**Figure 2:**
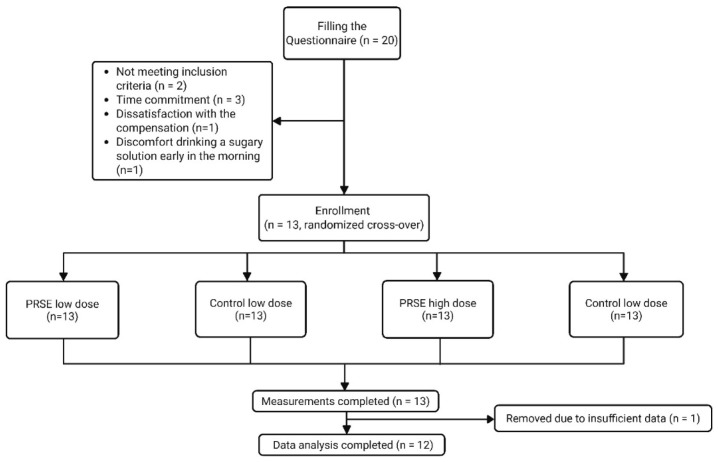
Consort flow diagram of participant inclusions. PRSE: (poly)phenol-rich sugarcane extract.

Table 1 lists the baseline characteristics of the 12 healthy subjects who completed the four-way intervention. One participant was excluded due to insufficient insulin data (Figure 2). The average age and BMI of the subjects were 25.5 ± 7.2 years and 23.6 ± 1.8 kg/m^2^. All participants had normal fasting blood glucose levels and regular blood pressure readings.

Table 2 presents the nutritional analysis of the (poly)phenol-free breakfast sandwich meal, with 1423.2 kJ/123.2 g consumed, equivalent to providing 50 g of carbohydrates, 11.8 g of protein, and 9.5 g of fat. The same breakfast meals were prepared similarly by one researcher (UHAJH) and provided along with the test drinks and water to the participants during each visit. No adverse reactions were reported following consumption of the breakfast meals or test drinks by any of the participants throughout the study.

### 3.2. Postprandial Blood Glucose Response and Plasma Insulin Response

**Figure 3:**
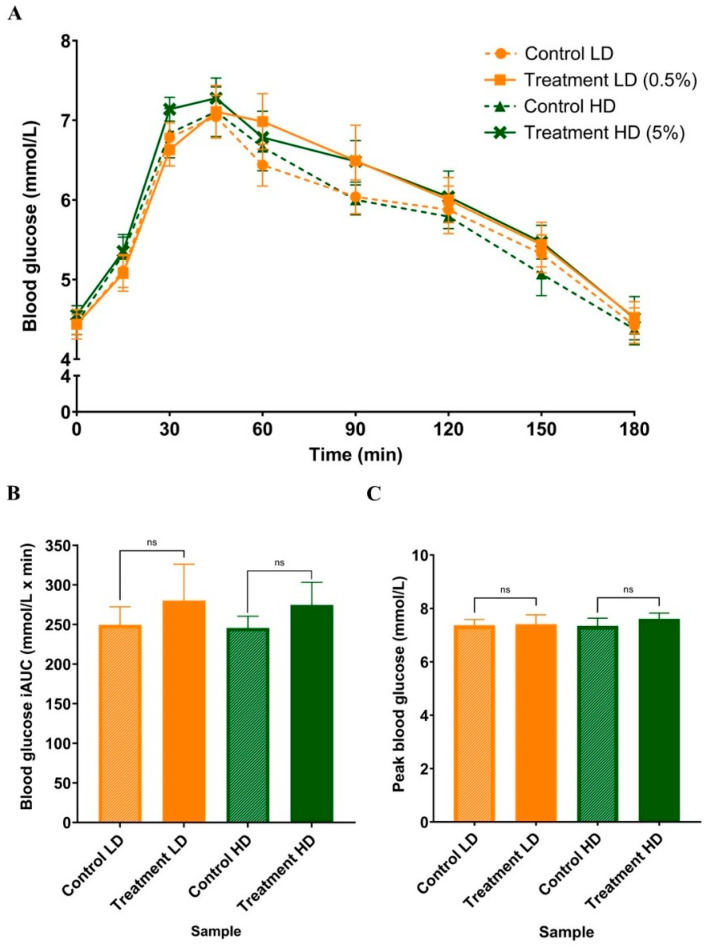
Comparison of mean blood glucose response at rest and in the postprandial state (**A**), iAUC_glucose_ (**B**) and peak blood glucose response (**C**) reported at 45 min, following consumption of 50 g of carbohydrate with sugarcane (poly)phenols at LD (0.5% *w*/*w*) and HD (5% *w*/*w*) and their corresponding controls. Data represent mean ± SEM (n = 13). LD: low-dose, HD: high-dose.

**Figure 4:**
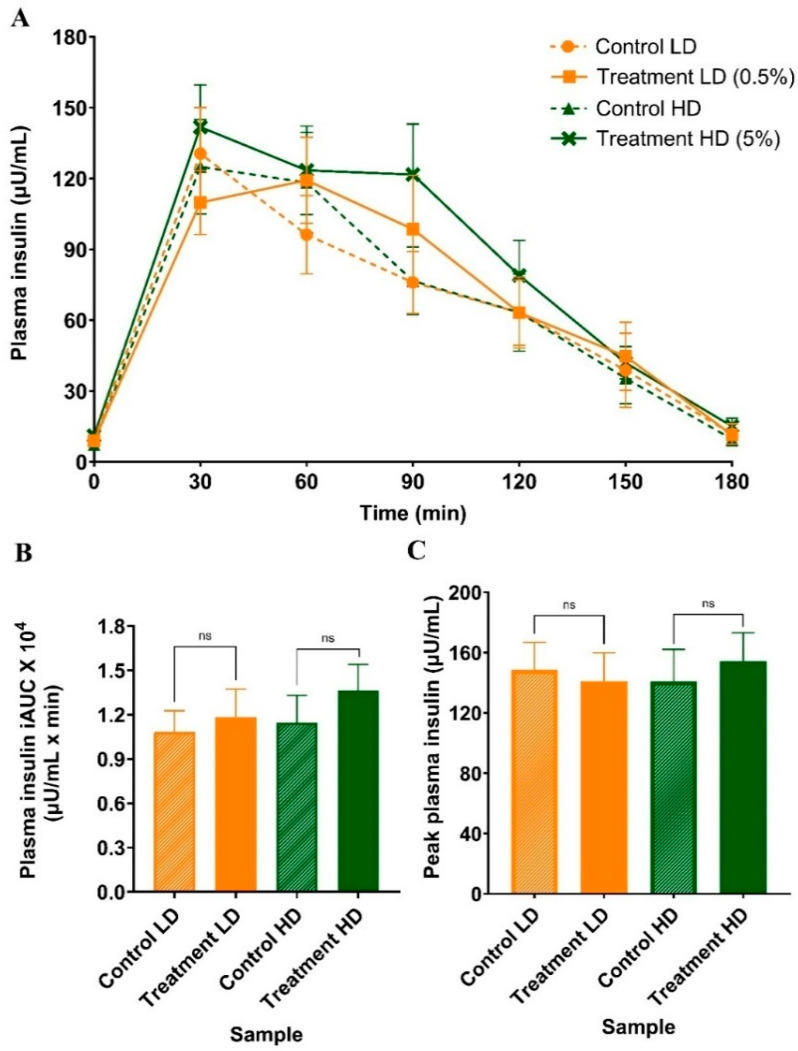
Comparison of mean plasma insulin response at rest and in the postprandial state (**A**), iAUC_insulin_ (**B**) and peak plasma insulin response (**C**) reported at 30 min except LD treatment, which was at 60 min, following consumption of 50 g of carbohydrate with sugarcane (poly)phenols at LD (0.5% *w*/*w*) or HD (5% *w*/*w*) and their corresponding controls. Data represent mean ± SEM (n = 13). LD: low-dose, HD: high-dose.

There were no significant Time × Trial differences in postprandial blood glucose (Figure 3A; *p* = 0.76), plasma insulin (Figure 4A; *p* = 0.39), total iAUC_glucose_ (Figure 3B; LD: *p* = 0.49, HD: *p* = 0.22), total iAUC_insulin_ (Figure 4B; LD: *p* = 0.42, HD: *p* = 0.07), peak blood glucose at 45 min (Figure 3C; LD: *p* = 0.83, HD: *p* = 0.11), and peak plasma insulin at 30–60 min (Figure 4C; LD: *p* = 0.52, HD: *p* = 0.38) between the interventions and controls. However, the insulin peak for the LD treatment, but not the HD, was delayed from 30 to 60 min (*p* = 0.05).

### 3.3. HOMA-IR Score and Matsuda Index

**Figure 5:**
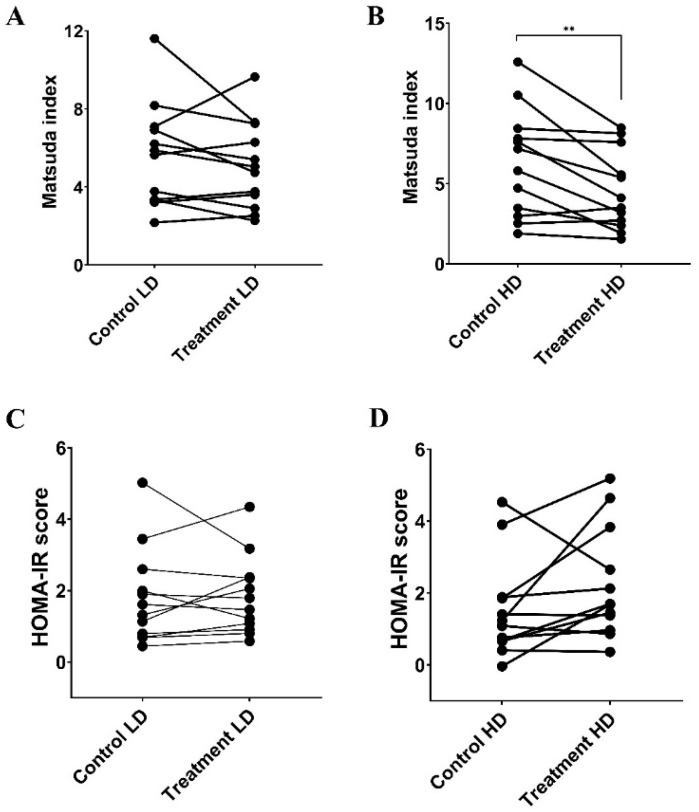
Paired comparison of MI and HOMA-IR score calculated using the glucose and insulin responses following consumption of 50 g of carbohydrate with sugarcane (poly)phenols at LD (0.5% *w*/*w*) (**A**,**C**) or HD (5% *w*/*w*) (**B**,**D**) and their corresponding controls. Data represent mean ± SEM (n = 12). ** *p* < 0.01. MI: Matsuda Index. HOMA-IR: Homeostatic Model Assessment for Insulin Resistance. LD: low-dose, HD: high-dose.

The calculated HOMA-IR scores were similar between LD (Figure 5C; *p* = 0.86) and HD (Figure 5D; *p* = 0.09) when compared to their corresponding controls. Interestingly, while the calculated MI did not differ between LD (MI: Figure 5A; *p* = 0.28) compared with its control, the high-dose MI was significantly lower than the corresponding control by 9.8% (Figure 5B; *p* = 0.007).

### 3.4. α-Glucosidase Enzyme Inhibition Assay

**Figure 6:**
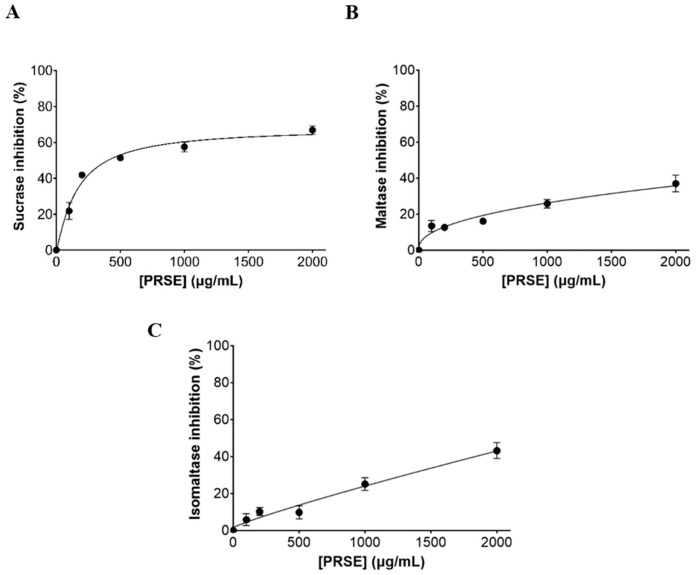
The inhibitory effect of sugarcane (poly)phenols extract (PRSE) on α-glucosidases extracted from human intestinal differentiated Caco-2/TC7 cell lysate (1 mg protein/mL). PRSE-S was tested at concentrations of 100–2000 µg/mL against sucrase (**A**), maltase (**B**) and isomaltase (**C**) activities using 20 mM corresponding substrates (sucrose, maltose, isomaltose). Results are from at least 2 sample injections into the HPAEC-PAD column, analyzed across 3 biological replicates of Caco-2/TC7 cell lysates (mean ± SEM). Where error bars are not visible, they are smaller than the data point.

PRSE inhibited human sucrase in a dose-dependent manner, maximal at 67% inhibition at the highest tested concentration of 2000 µg/mL, with an IC_50_ of 426 ± 19 µg/mL (Figure 6), compared to the positive control acarbose reported (IC_50_ of 1.51 ± 0.09 µg/mL) previously [18]. Enzyme inhibition by human maltase and isomaltase did not exceed ~40% even at the highest tested concentration of 2000 µg/mL, with IC_25_ values of 876 ± 131 µg/mL and 961 ± 95 µg/mL, respectively.

### 3.5. α-Amylase Inhibition Assay

**Figure 7:**
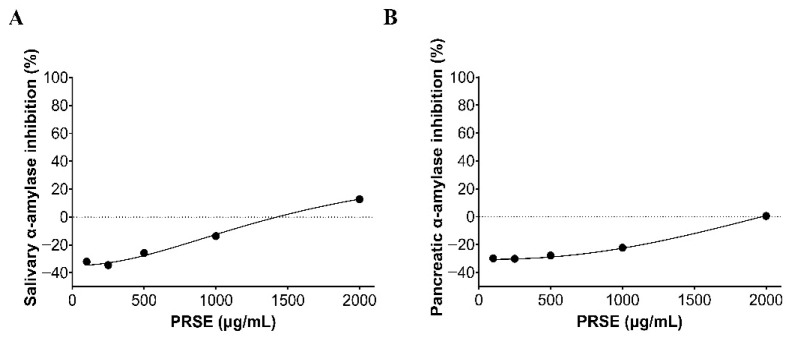
The inhibitory effect of sugarcane (poly)phenols extract (PRSE) on salivary (**A**) and pancreatic (**B**) α-amylases using Mal-7 (2 mM) as substrate. PRSE-S was tested at concentrations of 100–2000 µg/mL. Results are from at least 2 sample injections into the HPAEC-PAD column, analyzed across 3 replicates (mean ± SEM). Where error bars are not visible, they are smaller than the data point.

There was no significant inhibition of salivary α-amylase by PRSE at any concentration tested (Figure 7). Instead, concentrations below 1500 µg/mL and 2000 µg/mL appeared to enhance salivary and pancreatic α-amylase enzyme activity, respectively. At 2000 µg/mL, PRSE-S inhibited salivary α-amylase activity by ~12%, but with no effect on pancreatic α-amylase. The low concentration of free sugars in the extract was corrected in the control, and this did not affect the results.

### 3.6. Total (Poly)Phenol Content and (Poly)Phenol Composition of PRSE

Using the Folin–Ciocalteu method, the total (poly)phenol content was calculated as 23.5 ± 1.7 mg/mL (mean ± SEM, n = 3), equivalent to gallic acid as standard (R^2^ = 0.998, measured against 10 to 150 µg/mL gallic acid). Table 3 represents the (poly)phenols tentatively identified in both PRSE-L and PRSE-S used in both in vivo and in vitro parts of this study. The highest reported 10 peaks normalized to weight in PRSE-L were 4-hydroxybenzaldehyde, neochlorogenic acid/3-*O*-caffeoylquinic acid, vanilloloside, 3-coumaric acid, vanillin, 4-*O*-feruloyl-*D*-quinic acid, caffeic acid, 4-hydroxybenzoic acid, 3-*O*-feruloylquinic acid, gentisic acid and salicylic acid. The highest reported 10 peaks normalized to weight in PRSE-S were vanilloloside, neochlorogenic acid/3-*O*-caffeoylquinic acid, 3-*p*-coumaroylquinic acid, salicylic acid, 3-*O*-feruloylquinic acid (3FQA), caffeic acid, 4-hydroxybenzaldehyde, vanillin, (E)-3,4,5-trimethoxycinnamic acid, and 4-hydroxybenzoic acid.

As depicted in Table 3, the studied (poly)phenolic profiles are qualitatively similar between the different forms of PRSE with some quantitative differences. These small differences may slightly affect mechanistic interpretation of data when applied to the human study. However, the high sugar content (44.2%) of PRSE used in the human study (corrected for in the placebo) precluded its use in the enzyme assays, since the endogenous sugar would interfere in the assays even after blank correction.

## 4. Discussion

The randomized, single-blind, placebo-controlled, crossover study demonstrated that acute intake of PRSE at 0.5% (*w*/*w*) and 5% (*w*/*w*) has only mild effects on postprandial glycemia in healthy individuals following a sandwich breakfast meal consisting of 50 g of carbohydrate. Although postprandial glycemia decreased by 25% after a sucrose drink following a 32 mg (poly)phenol/100 g PRSE dose in healthy subjects [8], a similar effect was not observed here when healthy participants consumed a breakfast meal containing starch together with PRSE containing either 7.4 or 74 mg (poly)phenols per dose. In vivo results further explain why PRSE showed higher inhibition towards human sucrase activity than maltase or isomaltase activities seen in the in vitro analysis. Additionally, PRSE activated human α-amylases in the in vitro model, suggesting possible modest elevation of starch digestion.

There was a 30 min delay in the insulin peak after LD PRSE, but not after HD PRSE treatment. Although there was no statistically significant difference in the overall AUC or glycemic response, the delayed insulin–secretory response by PRSE warrants further exploration. A similar pattern has been observed during an intervention study using spinach extract (our unpublished data) and olive leaf powder where the insulin peak was delayed by 1 h [23] without any significant effect on glycemic response when tested in healthy human subjects. A more dramatic effect may be seen in metabolically compromised individuals or when higher concentrations are used where possible. Metabolically compromised individuals have early phase insulin secretion deficiencies due to poor β-cell function [24,25], and therefore, interventions that alter digestion/absorption, incretin signalling, oxidative stress, or insulin signalling, might significantly affect the insulin peak compared to healthy individuals who closely regulate fluctuations. Even though observed to a lesser extent, (poly)phenols have been demonstrated to lower peak insulin response and maintain insulin response, specifically when consumed with bread [26]. For instance, (poly)phenol-rich diets have shown improvements in glucose metabolism (i.e., reduced total plasma glucose AUC, increased early insulin secretion, improved post-challenge oral glucose sensitivity) in subjects that are at higher cardiometabolic risk [27]. Although no overall effects have been reported for type 2 diabetes patients, an acute cocoa supplementation showed a significant increase in overall serum insulin [28].

Although HOMA-IR is often used to assess insulin resistance, we did not observeany differences between treatments in an acute setting. It is speculated that food-based compounds consumed by healthy individuals are unlikely to show immediate changes or a substantial response after an acute intake [29]. Instead, the HD PRSE treatment significantly reduced the average calculated MI by 9.8%, relative to the matching control sample, suggesting a subtle alteration in the integrated glucose–insulin relationship, whereas LD treatment did not show a similar effect. MI is a whole-body insulin sensitivity index calculated from the simultaneous assessment of insulin and glucose levels after a carbohydrate load over 2 h, reported to be superior to the HOMA-IR for detecting insulin resistance or bodily insulin sensitivity [30]. This index predicts a composite of hepatic and peripheral insulin sensitivity during the basal state and after consumption. An MI below 4.0 is generally considered an insulin secretory defect in individuals with impaired glucose homeostasis, where peripheral insulin or glucose levels alone are insufficient to determine subtle changes in insulin secretion efficiency or glucose output [31]. Given the consistent carbohydrate intake across both trials, this may be attributed to the enhanced activity of the α-amylase enzyme as evidenced by in vitro data. Although postprandial glucose and insulin outcomes did not differ significantly between conditions, both were numerically higher following PRSE, consistent with a directional trend which may have affected MI in both mean glucose and insulin values, further reducing MI as a combined effect (Figure 7). Recently, nobiletin (a (poly)phenol in citrus fruit peels) was reported to enhance both human salivary and pancreatic α-amylases [17]. Nobiletin is not found in PRSE or other sugarcane products [3], and so we speculate that the effects are due to other (poly)phenol(s) or micronutrients found in sugarcane molasses, such as calcium and cobalt [3]. The aforementioned micronutrients have been shown to activate α-amylase enzyme activity [32,33] andenhance carbohydrate digestion, since α-amylase activity is dependent on a tightly bound calcium atom in the protein structure.

Our study is the first to report on inhibition of human α-glucosidase activities by sugarcane extracts of (poly)phenols. Sugarcane molasses have been reported to strongly inhibit yeast α-glucosidases [34,35], but this is irrelevant for determining effects in human digestive systems [36].

We speculate that stronger effects on postprandial glycemia may be observed in human interventions after longer-term PRSE supplementation. Previously, chronic intake of chlorogenic acid (a key compound in PRSE) has been shown to reduce fasting plasma glucose and insulin and improve insulin sensitivity in patients with impaired glucose tolerance [37]. In addition, a systematic analysis of studies on the efficacy of (poly)phenol-rich sources on acute postprandial glycemia found that only 6 out of 13 studies demonstrated a reduction in the postprandial glucose peak, and only 3 exhibited a decrease in the total AUC_glucose_ [26]. It is speculated that chronic consumption of (poly)phenols may provide cumulative protection to improve insulin secretion and overall glucose homeostasis.

Several limitations of this study should be noted. This includes the maximum tolerable concentration of PRSE when consumed with water, as higher concentrations were limited by taste and palatability, thereby limiting PRSE formulation to no more than 5% (*w*/*w*) to explore dose–response effects. We also tested the efficacy of PRSE on healthy individuals (despite varying BMI levels) who have unaltered postprandial glycemic control. Future studies should consider testing the efficacy of PRSE in at-risk populations with metabolic dysfunction after chronic consumption.

## 5. Conclusions

In summary, PRSE only mildly affected postprandial glucose and insulin responses in healthy individuals when consumed with a bread sandwich that contains starch. The differential effects of low- (delayed insulin peak) and high-dose PRSE (reduced MI potentially via activation of human α-amylases) warrant further exploration. The evidence observed in humans is consistent with the in vitro inhibitory potential of PRSE against human α-glucosidases (moderate to mild effects on sucrase, maltase and isomaltase) and activation of human α-amylases. Further investigation is necessary to clarify the chronic effects in a metabolically compromised population. The efficacy of PRSE may be more pronounced when consumed with sucrose than with complex starch-containing foods. This may open a few avenues for food innovation to supplement PRSE in sucrose-containing drinks rather than starch-containing solid foods.

## Figures and Tables

**Table 1:** Participant characteristics.

CHARACTERISTICS	MEAN ± SEM (N = 12)
**AGE (years)**	25.5 ± 7.2
**BMI (kg/m^2^)**	23.6 ± 1.8
**WAIST (cm)**	72.8 ± 4.3
**FAT MASS (kg)**	20.7 ± 3.8
**FAT FREE MASS (kg)**	42.9 ± 2.6
**SKELETAL MUSCLE MASS (kg)**	19.6 ± 1.5
**VISCERAL FAT (L)**	1.4 ± 0.3
**SYSTOLIC BLOOD PRESSURE (mmHG)**	110.8 ± 3.0
**DIASTOLIC BLOOD PRESSURE (mmHG)**	69.8 ± 2.1
**BASELINE BLOOD GLUCOSE LEVEL (mmol/L)**	4.5 ± 0.1

**Table 2:** Nutritional composition of the (poly)phenol-free breakfast sandwich meal.

Nutrient/Component	Value
Weight (g)	123.2
Energy (kJ)	1423.2
Protein (g)	11.8
Total fat (g)	9.5
Saturated fat (g)	3.7
Carbohydrate (g)	50.0
Sugars (g)	2.4
Starch (g)	47.6
Dietary fiber (g)	2.5
Sodium (mg)	441.3
Calcium (mg)	85.2
Energy from protein (%)	14.1
Energy from fat (%)	24.8
Energy from saturated fat (%)	9.6
Energy from carbohydrate (%)	59.6
Energy from fiber (%)	1.4
Total (poly)phenols (mg)	0.0

**Table 3:** (Poly)phenols tentatively identified in the liquid (used for human study) and powdered (used for enzyme assays) forms of PRSE.

Tentative Compounds Identified by Liquid Chromatography	Formula	MW	RT [min]	PRSE-L		PRSE-S	
				Weight Normalized Relative Peak Area × 10^6^	Relative Peak Area Normalized for 3FQA	Weight Normalized Relative Peak Area × 10^6^	Relative Peak Area Normalized for 3FQA
4-Hydroxybenzaldehyde	C_7_H_6_O_2_	122.04	5.78	225.4	533%	61.5	75%
Neochlorogenic acid/3-Caffeoylquinic acid	C_16_H_18_O_9_	354.09	11.55	93.7	222%	147.1	180%
Vanilloloside (glucovanillin)	C_14_H_20_O_8_	316.11	7.46	73.9	175%	169.6	208%
3-Coumaric acid	C_9_H_8_O_3_	164.04	6.64	72.1	171%	14.2	17%
Vanillin	C_8_H_8_O_3_	152.04	7.12	66.8	158%	45.3	55%
4-*O*-feruloyl-*D*-quinic acid	C_17_H_20_O_9_	368.11	22.71	64.7	153%	-	-
Caffeic acid	C_9_H_8_O_4_	180.04	8.79	49.2	116%	71.2	87%
4-Hydroxybenzoic acid	C_7_H_6_O_3_	138.03	4.15	43.0	102%	24.5	30%
3-*O*-Feruloylquinic acid (3FQA)	C_17_H_20_O_9_	368.11	21.02	42.3	100%	81.7	100%
Gentisic acid	C_7_H_6_O_4_	154.02	2.70	33.9	80%	17.4	21%
Salicylic acid	C_7_H_6_O_3_	138.03	12.97	28.1	67%	104.3	128%
3-*p*-Coumaroylquinic acid	C_16_H_18_O_8_	338.10	17.68	26.0	62%	121.3	148%
(E)-3,4,5-Trimethoxycinnamic acid	C_12_H_14_O_5_	238.08	18.92	17.1	41%	33.3	41%
Isovitexin2″-*O*-arabinoside	C_26_H_28_O_14_	564.14	23.46	15.5	37%	1.86	2%
Isoferulic acid/3-Hydroxy-4-methoxycinnamic acid	C_10_H_10_O_4_	194.05	17.06	12.2	29%	11.7	14%
3-Methoxy-5,7,3′,4′-tetrahydroxy-flavone	C_16_H_12_O_7_	316.05	25.36	11.5	27%	14.5	18%
2-Anisic acid	C_8_H_8_O_3_	152.04	9.27	8.11	19%	3.67	4%
Trans-5-*O*-(4-coumaroyl)-*D*-quinic acid/ 2-coumaroylquinic acid	C_16_H_18_O_8_	338.10	19.38	7.19	17%	20.7	25%
Retusin/quercetin-3,7,3′,4′-tetramethyl ether	C_19_H_18_O_7_	358.10	22.13	5.80	14%	4.80	6%
Rhapontigenin	C_15_H_14_O_4_	258.08	28.01	5.12	12%	3.48	4%
Isofraxidin	C_11_H_10_O_5_	222.05	19.94	5.12	12%	3.66	4%
Salidroside	C_14_H_20_O_7_	300.12	17.85	5.10	12%	9.94	12%
Luteolin 6-*C*-glucoside 8-*C*-arabinoside	C_27_H_30_O_16_	610.15	23.18	5.03	12%	3.79	5%
D-(-)-Quinic acid	C_7_H_12_O_6_	192.06	22.71	4.76	11%	7.29	9%
Syringic acid	C_9_H_10_O_5_	198.05	6.60	3.44	8%	2.24	3%
Luteolin	C_15_H_10_O_6_	286.04	27.99	2.70	6%	0.64	1%
Sinapinic acid	C_11_H_12_O_5_	224.06	18.17	2.20	5%	3.16	4%
3,4-Dihydroxyphenylacetic acid (homoprotocatechuic acid)	C_8_H_8_O_4_	168.04	2.43	1.99	5%	-	-
Ferulic acid 4-glucuronide	C_16_H_18_O_10_	370.08	11.24	1.91	5%	5.06	6%
Vanillic acid	C_8_H_8_O_4_	168.04	7.73	1.08	3%	1.58	2%
Catechin	C_15_H_14_O_6_	290.07	18.08	0.74	2%	0.84	1%
Gallic acid	C_7_H_6_O_5_	170.02	1.66	0.46	1%	0.10	0%
Apigenin-7-*O*-glucoside	C_21_H_20_O_10_	432.10	24.39	0.46	1%	-	-
Rutin	C_27_H_30_O_16_	610.15	25.11	0.12	0%	0.01	0%

## Data Availability

The original contributions presented in this study are included in the article. Further inquiries can be directed to the corresponding author.

## Abbreviations

The following abbreviations are used in this manuscript:

AUC	Area Under the Curve
BMI	Body Mass Index
CFE	Cell Free Extract
DNSA	3,5-Dinitrosalicylic acid
ELISA	Enzyme-Linked Immunosorbent Assay
FODMAP	Fermentable Oligosaccharides Disaccharides Monosaccharides and Polyols
HD	High Dose
HOMA-IR	Homeostasis Model Assessment for Insulin Resistance
HPAEC-PAD	High-Performance Anion-Exchange Chromatography with Pulsed Amperometric Detection
iAUC	incremental Area Under the Curve
LD	Low Dose
Mal-7	Maltoheptaose
MI	Matsuda Index
PBS	Phosphate Buffer Solution
PRSE	(Poly)phenol-Rich Sugarcane Extract
PRSE-L	(Poly)phenol-Rich Sugarcane Extract-Liquid
PRSE-S	(Poly)phenol-Rich Sugarcane Extract-Solid
